# Prevalence and associated factors of needlestick and sharp object injuries among healthcare workers in Ethiopia: a systematic review and meta-analysis

**DOI:** 10.3389/fepid.2024.1385417

**Published:** 2024-06-25

**Authors:** Gudeta Kaweti, Tihun Feleke

**Affiliations:** ^1^ Department of Social and Population Health, Yirgalem Hospital Medical College, Yirgalem, Ethiopia; ^2^ Hawassa College of Health Science, Research and Community Service Directorate, Hawassa, Ethiopia

**Keywords:** Ethiopia, needlestick injury, sharp objects injury, pooled prevalence, systematic review, meta-analysis

## Abstract

**Background:**

Needlestick and sharp object injuries affect healthcare workers. However, there are limitations in the evidence available for informed decision-making by stakeholders, as individual research shows inconsistent results. Therefore, this study aims to assess the pooled prevalence of needlestick and sharp object injuries and their associated factors.

**Methods:**

MEDLINE/PubMed, EMBASE, Web of Science, Google Scholar, and other databases were searched from 5 September 2023 to 10 October 2023 using the following search terms: “Prevalence” OR “Burden” OR “Magnitude” AND “Associated factors” OR “related factors” OR “Risk factors” OR “determinants” OR “Predictors” AND “Needle stick Injury” OR “Sharp Injury” OR “Health care Workers” OR “ Health Care Personnel” OR “Nurses” OR “Professional” AND “Ethiopia”.

**Results:**

The pooled prevalence of needle sticks and sharp objects injury was 40.5 (95% CI: 35.0, 45.9). Needle-stick (AOR, 2.3; 95% CI: 1.6, 3.3, *P* < 0.001], absence of routine precaution [AOR, 2.3; 95% CI: 1.1, 4.5, *P* < 0.01] and lack of training (AOR = 2.4; 95% CI: 1.4, 4.1, *p* < 0.001) had increased odds of needle-sticks and sharp objects injury.

**Conclusion:**

Forty percent of healthcare workers in Ethiopia have experienced needlestick and sharp object injuries. The identified factors included recapping, absence of routine precautions, and lack of training.

**Systematic Review Registration:**

PROSPERO, identifier (CRD42023462311).

## Introduction

Needlestick and sharp object injuries affect healthcare workers (HCWs) in both industrialized and developing nations, although their severity and extent may vary. The World Health Organization (WHO) estimates that illnesses related to the workplace account for around 11% of the global disease burden (1). In US hospitals, an estimated 385,000 injuries occur annually (2). The burden is significantly more in the facilities of the countries of sub-Saharan Africa, including Ethiopia (3–5). The rapid expansion of service delivery and production industries in Ethiopia needs adequate attention to protect the health and safety of HCWs. Poor-quality equipment, substandard work practices, and a high burden of communicable diseases force the country to do so. The Awareness status, nature of medical devices, handling practices for sharp objects, and use of personal protective equipment (PPE) are among the major determinants for exposure to needlestick and sharp object injuries (6, 7).

Healthcare workers may sustain injuries during blood sample collection, surgical procedures, intravenous line administration, needle recapping, and due to inadequate waste disposal methods (8). The greatest risk, following needlestick and sharp object injuries, is the transmission of blood-borne infections from patients to HCWs (4). The type of injury, the amount of blood delivered to the HCWs during the exposure, the immunological status of the worker, and source patient factors all influence the risk of transmission (9). Globally, 3 million HCWs are exposed to blood-borne viruses annually; of these, 2 million are linked to HBV, 900,000 are linked to HCV, and 300,000 are linked to HIV (10). The risk of acquiring HBV, HCV, and HIV infections via needlestick and sharp object injuries can range from 2% to 40%, 3% to 10%, and 0.2% to 0.5%, respectively, if the source patient is positive (11, 12).

As far as we know, there is no nationally representative empirical study finding on the prevalence and associated factors of needlestick and sharp object injuries among HCWs in Ethiopia. However, numerous scattered and limited research studies have been carried out to determine the magnitude of needlestick and sharp object injuries and associated factors (13). One published study in 2019 had limited scope since it only focused on needlestick injuries, excluding injuries due to other sharp objects (14). The main objective of this systematic review and meta-analysis is therefore to estimate the national pooled prevalence and associated factors of needlestick and sharp object injuries. The results of the study will be used to provide nationwide insight for occupational health and safety policymakers and implementers to inform, plan, implement, and evaluate their strategies.

## Objectives

The aim of this study is to assess the pooled prevalence of needlestick and sharp object injuries and associated factors among healthcare workers in Ethiopia.

### Research questions

1. What is the pooled prevalence of needlestick and sharp object injuries among healthcare workers in Ethiopia?
2. What are the determinants of needlestick and sharp object injuries among healthcare workers in Ethiopia?

## Methods

### Study review protocol

This study was conducted in accordance with the guidelines of Preferred Reporting Items for Systematic reviews and Meta-Analyses (PRISMA). The protocol was registered in the international prospective register of systematic reviews (PROSPERO) under an identification code **CRD42023462311**.

### Inclusion criteria

Full-text papers published in English from Ethiopian healthcare facilities that passed quality assessment standards and reported both needlestick and sharp object injuries were included regardless of their study design and publication time. These included studies were conducted before 10 October 2023.

**Population (P)** stands for healthcare workers/healthcare providers/health professionals, while **intervention or exposure (I)** stands for determinants/predictors/factors affecting needlestick and sharp object injuries. **Comparison (C)** is not applicable, while the **outcome (O)** of the study was the pooled prevalence of needlestick and sharp object injuries among healthcare workers.

### Exclusion criteria

Studies that were not fully accessed, had difficulty in data extraction, had irretrievable full texts, and did not properly report the outcome of interest were excluded. In addition, articles with unrelated outcome measures or those with missing or insufficient outcomes were also excluded.

### Selection of studies

All search results were exported to the EndNote X7 citation manager, and duplicates were removed. Articles were then screened and evaluated independently by authors GK and TF through a careful reading of the title and abstract. The titles and abstracts of studies that clearly mentioned the outcomes of the review were considered for further evaluation.

**Figure 1:**
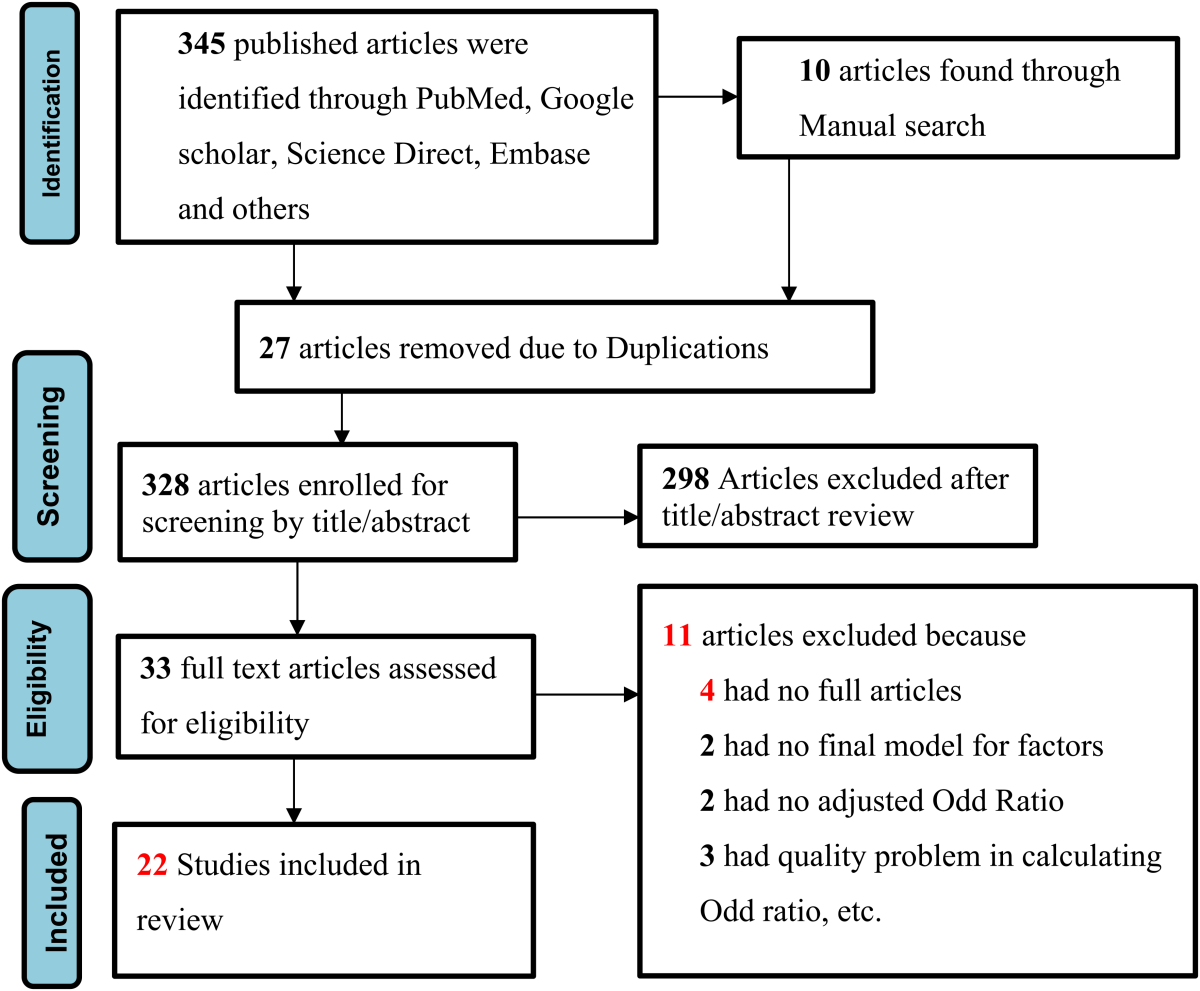
Flowchart of selected articles included in this systematic review and meta-analysis conducted in Ethiopia in 2023.

The full texts of the retained studies were further evaluated by the authors against the Joanna Briggs Institute (JBI) critical appraisal tool for cross-sectional study checklists based on the quality of their objectives, methods, participants/populations, and key findings. In case of disagreement between the authors, the differences were resolved by consensus for the inclusion. The overall study selection process is presented using the PRISMA statement flow diagram (Figure 1).

### Search engines

Major databases such as MEDLINE/PubMed, EMBASE, Web of Science, and others, including Google Scholar, were searched to identify information that was pertinent and closely related to the variables of interest.

### Searching strategies

The authors used selected keywords and MeSH terms with Boolean logic operators (AND, OR), both individually and in conjunctions, by extracting them from the review questions as follows. The search strategy included the following keywords: “Prevalence” OR “Burden” OR “Magnitude” AND “Associated factors” OR “related factors” OR “Risk factors” OR “determinants” OR “Predictors” AND “Needle stick Injury” OR “Sharp Injury” OR “Health care Workers” OR “ Health Care Personnel” OR “Nurses” OR “Professional” AND “Ethiopia”. A manual search of various repositories was conducted to retrieve unpublished studies. The overall search result was compiled using EndNote X7 citation manager software. The search was conducted between 5 September 2023 and 10 October 2023.

### Data extraction

The authors (GK and TF) independently extracted data using the standardized data extraction format adapted from the JBI. The format developed for the first objective included the name of the first author with publication year, region, study design, sample size, and study response rate.

The data extraction format for the second objective included the most frequently reported predictors of percutaneous injuries. The included predictors were sex (male or female), recapping (yes or no), using personal protective equipment (yes or no), training (yes or no), and working hours per week (40 and below 40 h or more than 40 h).

### Outcome measurements

Two objectives were addressed by this systematic review and meta-analysis. The first objective of this study was to assess the pooled prevalence of needlestick and sharp object injuries among healthcare workers in Ethiopia. The second objective was to determine the pooled effects of factors associated with needlestick and sharp object injuries among healthcare workers in Ethiopia.

### Quality assessment and risk of bias

The JBI’s critical appraisal checklist, developed for analytical cross-sectional study, was applied to evaluate the reliability and methodological validity of the included studies. The checklist has eight criteria that are measured with the options “Yes,” “No,” “Unclear,” and “Not applicable.” We classified “Yes” scores below 50%, 50%–80%, and more than 80% for each JBI criterion as indicating high, medium, and low publication bias, respectively.

To minimize bias, the authors (GK and TF) independently screened the studies. Funnel plot asymmetry and Egger's test were used to check for publication bias. After running Egger's test, a *p*-value of less than 0.05 as a cutoff point was used to declare the presence of publication bias.

## Results

### Search results

This study covers the period from 5 September 2023 to 10 October 2023. A total of 355 articles were identified through electronic database search (345 articles) and manual search (10 articles), of which 27 articles were excluded due to duplication. Subsequently, 298 primary publications were excluded after a review of their titles and abstracts revealed that they were conducted in other contexts and were not relevant to our study. We removed another 11 articles for other reasons and finally included 22 primary research articles for the study, based on our eligibility criteria, after reading their entire texts (Figure 1).

### Characteristics of included articles

All 22 original articles that were included were cross-sectional studies. The sample sizes ranged from 112 (15) to 760 (16), giving a total sample size of 8,087 participants. The response rate ranged from 82% (16) to 100% (17), with an average of 94.17%. Among the included original articles, eight studies were conducted in Amhara regional state (17–24), two were conducted in the Addis Ababa city administration (25, 26), four studies each were conducted in the Oromia regional state (27–30) and the South Nations, Nationalities, and Peoples’ Region (SNNPR) (15, 16, 31), and one study each was conducted in the Tigray regional state (32), Dire Dawa (33), Gambella (34), Sidama (35), and Somali (36) (Table 1).

**Table 1:** List of included studies with their key characteristics conducted in Ethiopia, 2023.

S. No.	Authors name	Study area	Design	Sample size	Success	Prevalence	Lower CL	Upper CL
1	Abadiga et al. (2020)	Oromia	Cross sectional	310	100	**32.26**	27.05	37.46
2	Abebe et al. (2018)	Amhara	Cross sectional	158	65	**41.14**	33.47	48.81
3	Adola (2020)	Oromia	Cross sectional	383	166	**43.34**	38.38	48.31
4	Afework et al. (2023)	SNNPR	Cross sectional	112	64	**57.14**	47.98	66.31
5	Alemayehu et al. (2022)	Amhara	Cross sectional	362	131	**36.19**	31.24	41.14
6	Assen et al. (2020)	Amhara	Cross sectional	457	124	**27.13**	23.06	31.21
7	Bazie (2020)	Amhara	Cross sectional	362	203	**56.08**	50.96	61.19
8	Bekele et al. (2015)	Oromia	Cross sectional	362	126	**34.81**	29.90	39.71
9	Berhan et al. (2021)	Amhara	Cross sectional	318	87	**27.36**	22.46	32.26
10	Beyene et al. (2021)	Addis Ababa	Cross sectional	297	96	**32.32**	27.00	37.64
11	Bidiri et al. (2014)	Oromia	Cross sectional	232	83	**35.78**	29.61	41.94
12	Dilie et al. (2016)	Amhara	Cross sectional	213	36	**16.90**	11.87	21.93
13	Getie et al. (2020)	Amhara	Cross sectional	147	111	**75.51**	68.56	82.46
14	Kaweti & Abegaz (2016)	Sidama	Cross sectional	526	226	**42.97**	38.74	47.20
15	Kebede et al. (2016)	Amhara	Cross sectional	313	123	**39.30**	33.89	44.71
16	Liyew et al. (2020)	Addis Ababa	Cross sectional	275	97	**35.27**	29.63	40.92
17	Mengistu et al. (2020)	Gambella	Cross sectional	362	113	**31.22**	26.44	35.99
18	Mekonnen et al. (2018)	Dire Dawa	Cross sectional	305	149	**48.85**	43.24	54.46
19	Tadesse et al. (2016)	SNNPRs	Cross sectional	760	396	**52.11**	48.55	55.66
20	Tsegayeamlak et al. (2023)	SNNPRs	Cross sectional	341	104	**30.50**	25.61	35.39
21	Weldesamuel et al. (2019)	Tigray	Cross sectional	456	171	**37.50**	33.06	41.94
22	Yeshitila et al. (2015)	Somali	Cross sectional	660	389	**58.94**	55.19	62.69

*CL, confidence level.

### Types of injuries

The attempt to classify injuries by needlesticks and other sharp objects to check the burden areas of the problem was unsuccessful due to the absence of clear classifications in the included studies (Table 2).

**Table 2:** Characteristics of injuries for the included studies conducted in Ethiopia, 2023.

S. No.	Authors name	Sample size	Injuries			
			Ever	Last 12 months	Needle sticks	Sharp objects
1	Abadiga et al. (2020)	310	100	100	Ns	Ns
2	Abebe et al. (2018)	158	65	65	Ns	Ns
3	Adola (2022)	383	166	71	Ns	Ns
4	Afework et al.	112	64	NI	33	31
5	Alemayehu et al. (2022)	362	131	86	Ns	Ns
6	Assen et al. (2020)	457	124	124	Ns	Ns
7	Bazie (2020)	362	203	143	Ns	Ns
8	Bekele et al. (2015)	362	134	26	Ns	Ns
9	Berhan et al. (2021)	318	87	87	Ns	Ns
10	Beyene et al. (2021)	297	96	96	96	Ns
11	Bidiri et al. (2014)	211	83	83	83	Ns
12	Dilie et al. (2016)	213	36	36	26	8
13	Getie et al. (2020)	147	111	111	46	65
14	Kaweti et al. (2016)	526	226	64	Ns	Ns
15	Kebede et al. (2018)	313	123	87	Ns	Ns
16	Liyew et al. (2020)	275	97	97	Ns	Ns
17	Mengistu et al. (2020)	362	113	113	Ns	Ns
18	Mekonnen et al. (2018)	305	149	75	Ns	Ns
19	Tadesse et al. (2016)	760	396	343	Ns	Ns
20	Tsegaye Am et al. (2023)	341	104	Ns	Ns	Ns
21	Weldesamuel et al. (2019)	456	171	Ns	Ns	Ns
22	Yeshitila et al. (2015)	660	389	377	Ns	Ns

Note: Ns means not specified

### Overall quality of included studies

Based on the JBI critical appraisal checklist, the overall quality of the studies was supposed to be 22 × 8 = 176. Based on our evaluation, the quality score was 83.5% (147/176 × 100), which met the JBI requirement (Table 3).

**Table 3:** Overall paper quality of eligible studies based on nine statements of JBI.

S. No.	Statement of JBI for identified studies (*n* = 22)	Total yes (x/22)	%
1	Were the criteria for inclusion in the sample clearly defined?	15	68.2
2	Were the study subjects and the setting described in detail?	17	74
3	Was the exposure measured in a valid and reliable way?	19	86.4
4	Were objective, standard criteria used for measurement of the condition?	20	87
5	Were confounding factors identified?	14	61
6	Were strategies to deal with confounding factors stated?	20	87
7	Were the outcomes measured in a valid and reliable way?	21	91
8	Was appropriate statistical analysis used?	19	83
	Overall Evaluation	145	82.4

### Meta-analysis

**Figure 2:**
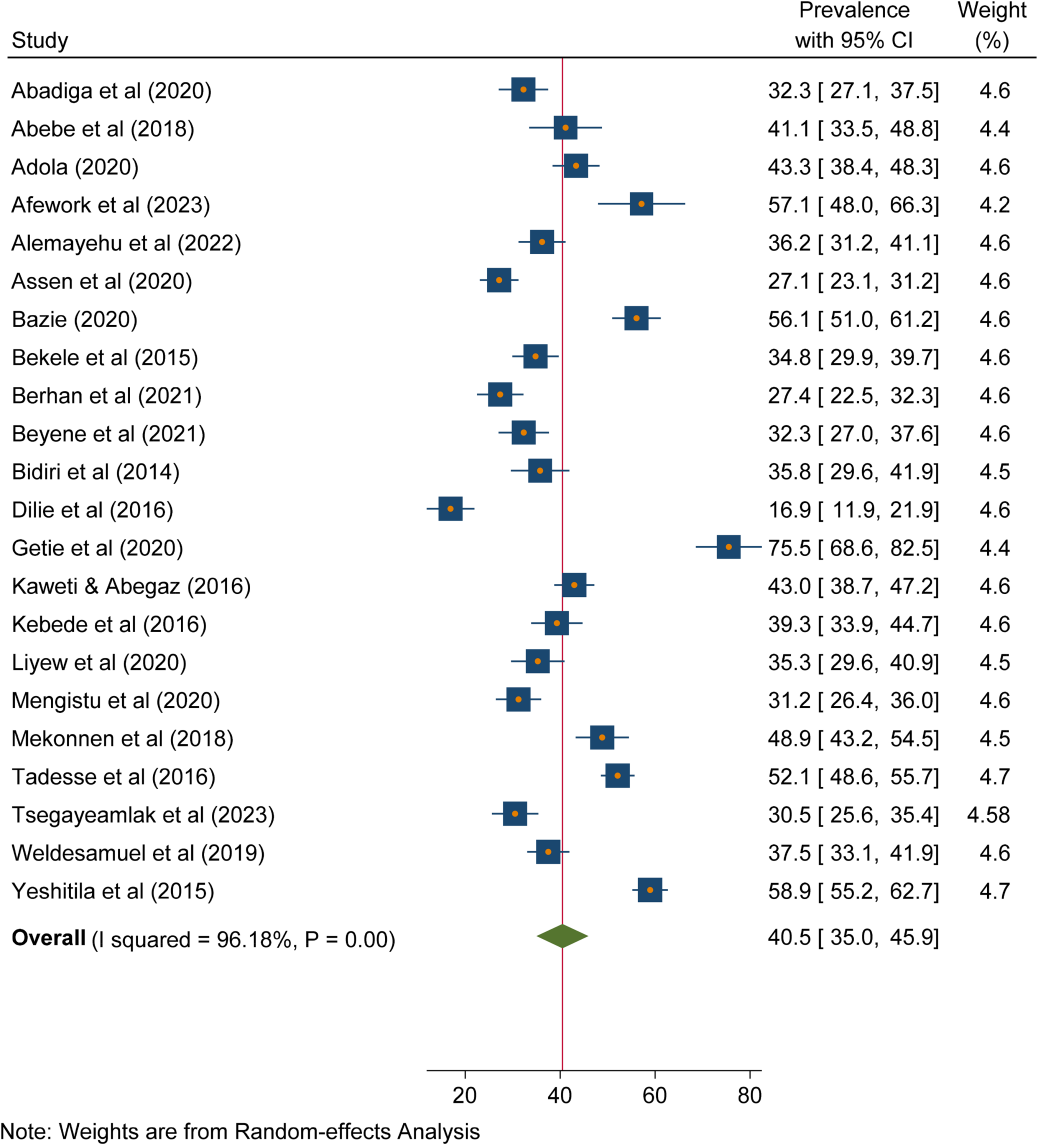
Forest plot of the included studies to determine the pooled prevalence of needlestick and sharp object injuries among healthcare workers in Ethiopia, 2023.

The forest plot below indicated that the pooled prevalence of needlestick and sharp object injuries among healthcare workers in Ethiopia was 40.5 (95% CI: 35.0, 45.9). With regard to heterogeneity, *I*^2^ was determined, and there was high heterogeneity as evidenced by *I*^2^ = 96.18% and *p* < 0.001. Therefore, a random effects model was used to estimate the pooled prevalence of needlestick and sharp object injuries among healthcare workers in Ethiopia (Figure 2).

### Publication bias

**Figure 3:**
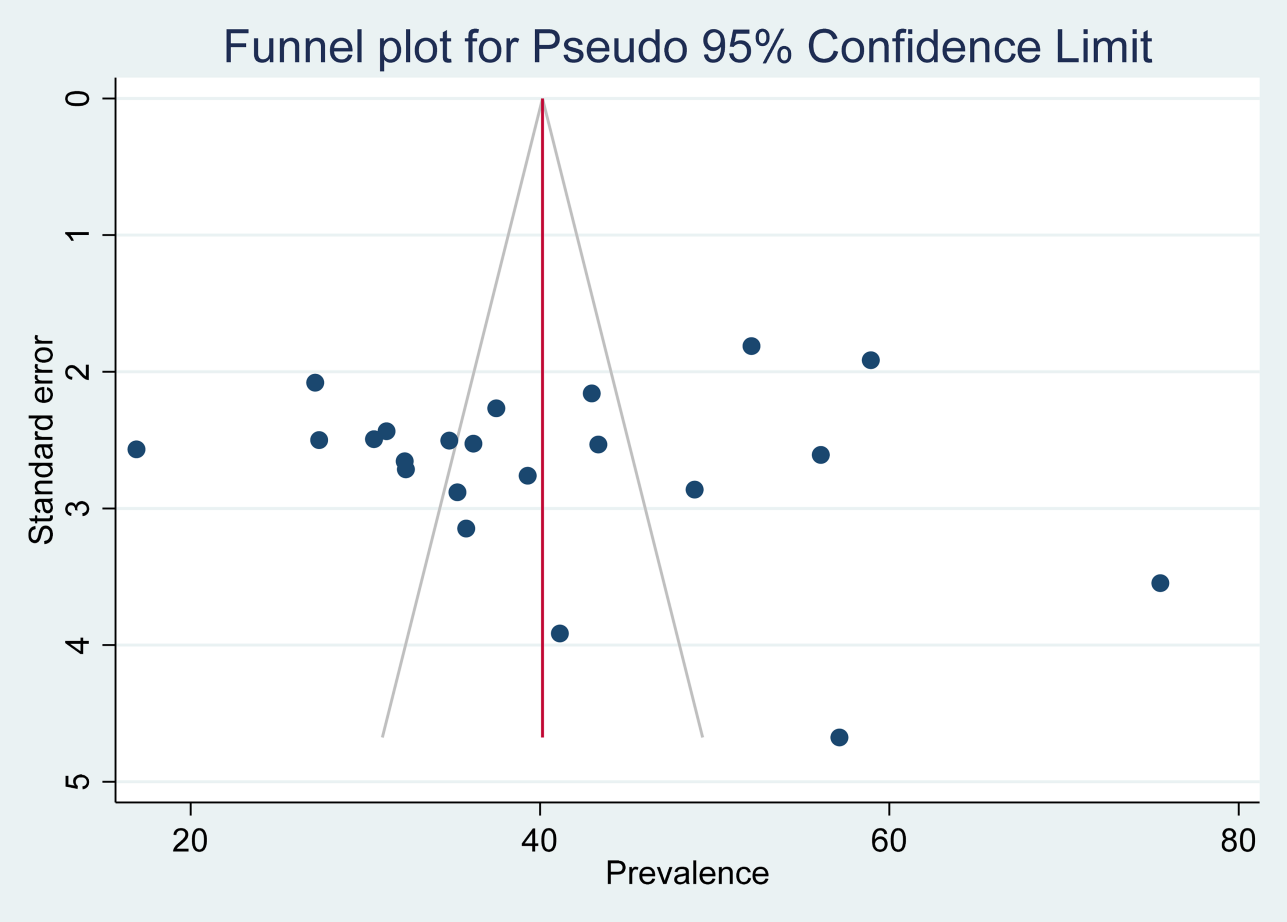
Funnel plot with pseudo-95% CI for the prevalence of needlestick and sharp object injuries in Ethiopia, 2023.

Publication bias was detected, as we can observe in the funnel plot in Figure 3. In addition, Eggers’ tests were done, and there was evidence for publication bias with a *p*-value of 0.4626.

### Contributing factors

This systematic review and meta-analysis was not limited to the study of the pooled prevalence of needlestick and sharp object injuries. We reviewed contributing factors for needlestick and sharp object injuries using the adjusted odds ratio (AOR) of the included articles. That means that we excluded articles with crude odd ratios but without adjusted odd ratios.

### Sex of the participants

**Figure 4:**
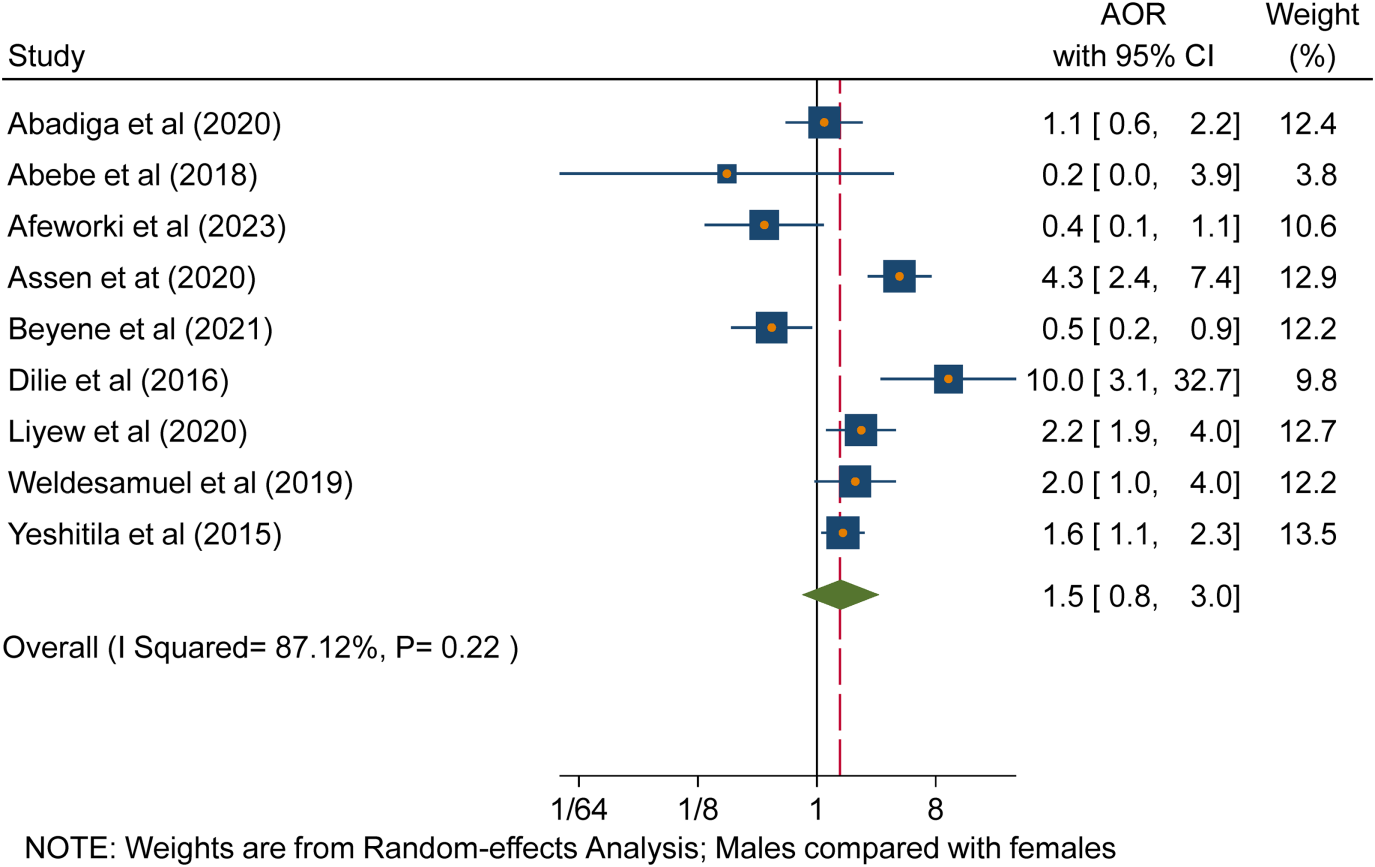
Forest plot indicating the association between sex and needlestick and sharp object injuries in Ethiopia, 2023.

There is no statistically significant association between the sex of the healthcare workers and the occurrence of needlestick and sharp object injuries in Ethiopia (Figure 4). This figure indicates the odds of a male's exposure to needlestick and sharp object injuries compared to a female’s.

### Needlestick recapping

**Figure 5:**
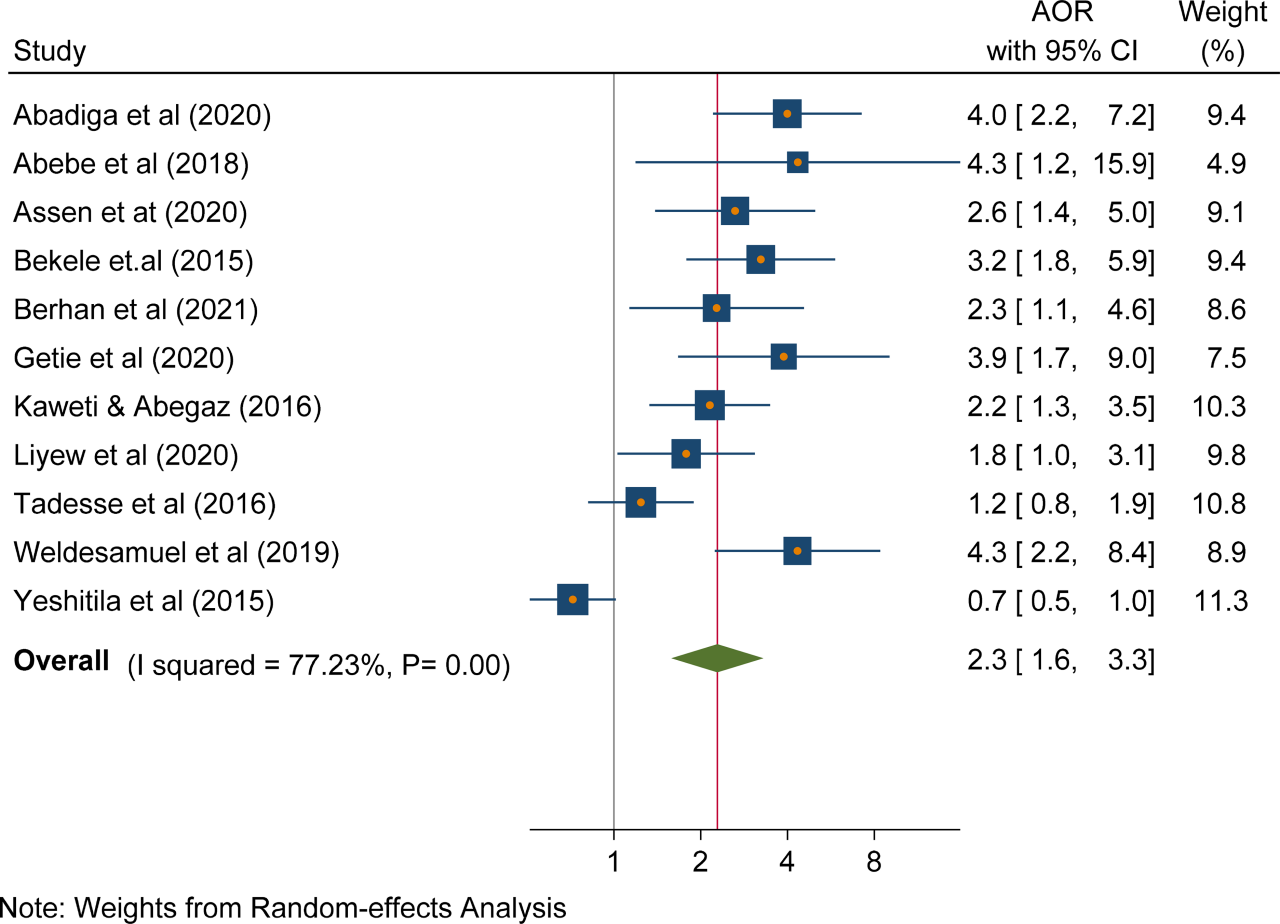
Forest plot indicating the association between needlestick recapping and needlestick and sharp object injuries in Ethiopia, 2023**.**

According to the findings of this review, there was a statistically significant association between needlestick recapping and needlestick and sharp object injuries (AOR, 2.3; 95% CI: 1.6, 3.3, *P*<0.001]. Accordingly, needle-stick recapping increases the odds of needle sticks and sharp objects injuries by 2.3 folds. A heterogeneity test indicated an *I*^2^ of 77.23% (Figure 5).

### Standard precautions

**Figure 6:**
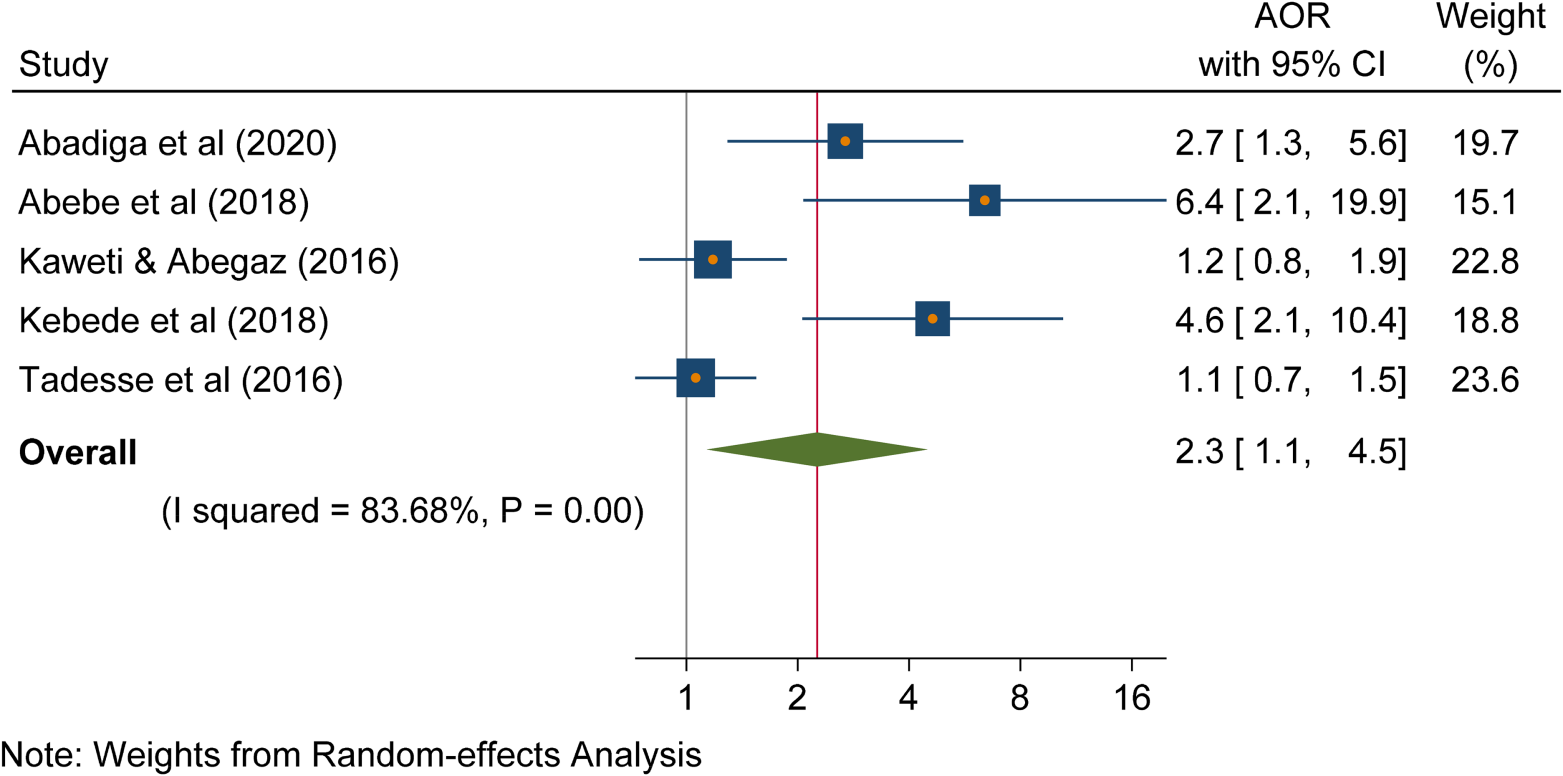
Forest plot indicating the association between following standard precautions and needlestick and sharp object injuries in Ethiopia, 2023.

According to the results of this review, there was a statistically significant association between the absence of routine follow-up of standard precautions and needlestick and sharp object injuries (AOR, 2.3; 95% CI: 1.1, 4.5, *p* < 0.01). Healthcare workers who did not follow standard precautions were 2.2 times more likely to suffer needlestick and sharp object injuries compared to their remaining counterparts. A heterogeneity test indicated an *I*^2^ of 83.63% (Figure 6).

### Training

**Figure 7:**
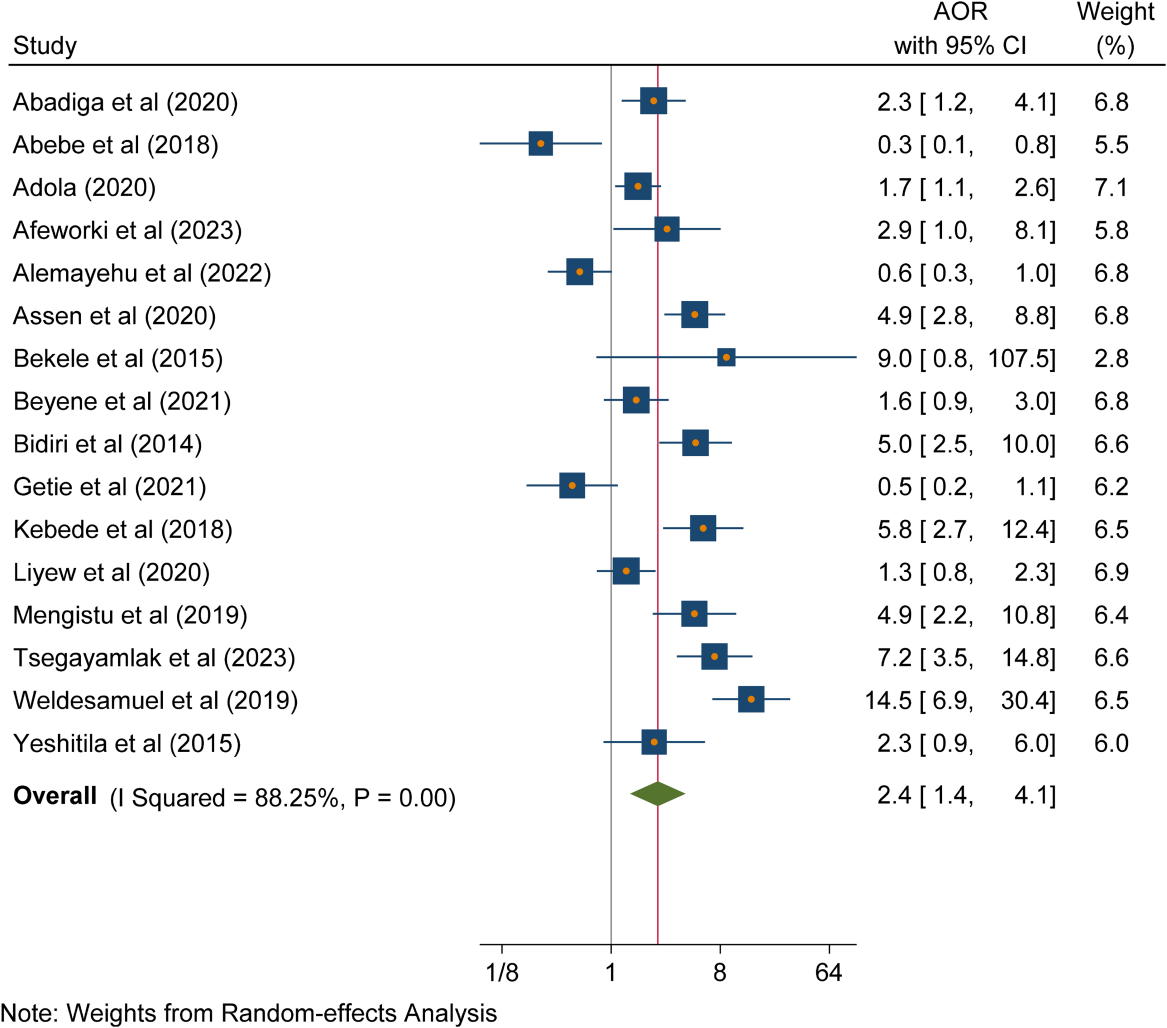
Forest plot indicating the association between training and needlestick and sharp object injuries in Ethiopia, 2023.

The findings of the review indicated a significant association between training and needlestick and sharp object injuries. Healthcare workers who had not received training were 2.41 times more likely to be exposed to needlestick and sharp object injuries compared to healthcare workers who had been trained on injuries (AOR = 2.4; 95% CI: 1.4, 4.1, *p* < 0.001). A heterogeneity test indicated an *I*^2^ =  88.25% (Figure 7).

### Working more than 40 h per week

**Figure 8:**
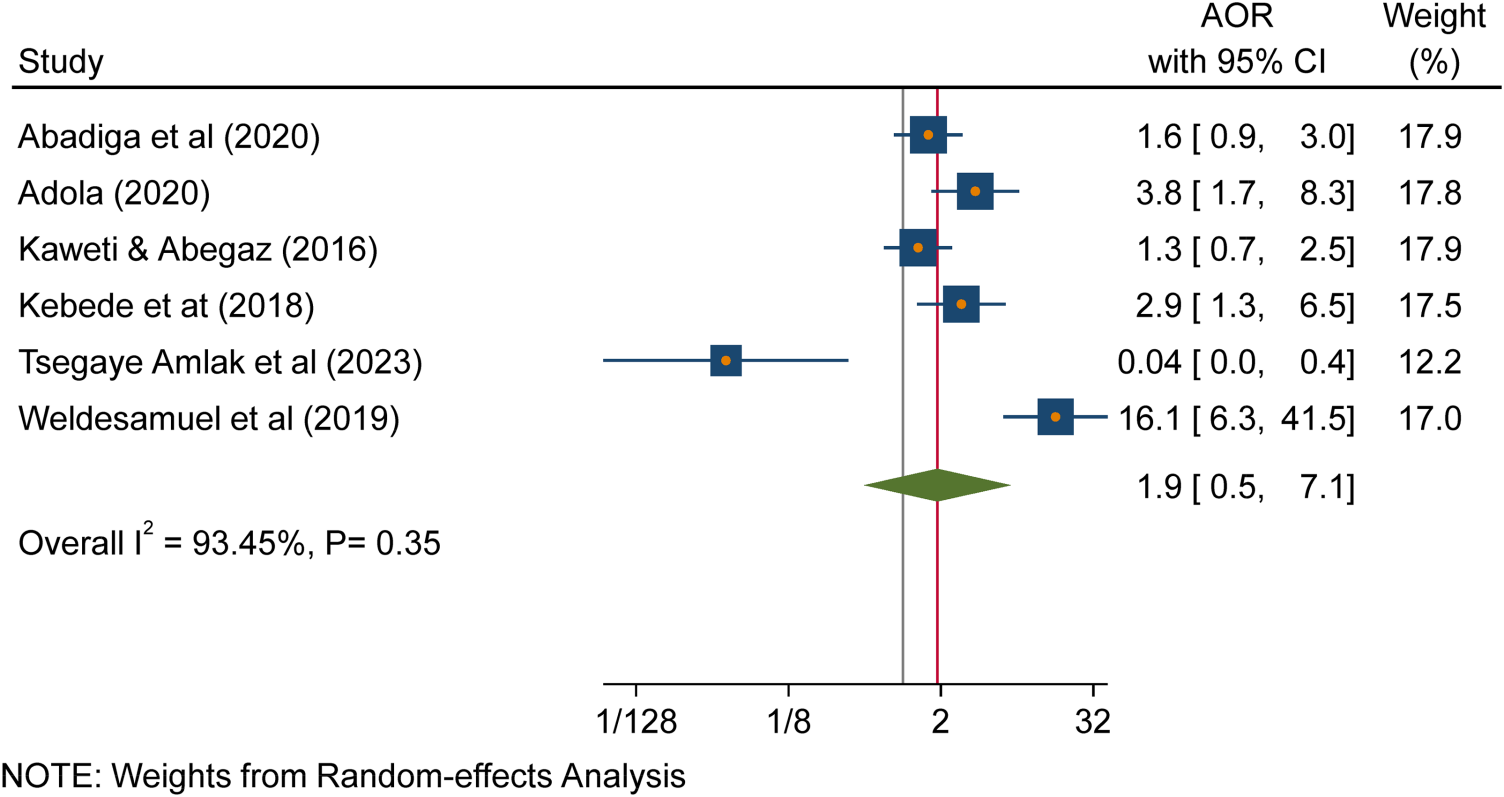
Forest plot indicating the association between working hours and needlestick and sharp object injuries in Ethiopia, 2023.

According to the findings of this study, there is no association between working hours and needlestick and sharp object injuries (Figure 8).

## Discussion

The aim of this systematic review and meta-analysis was to investigate the burden of needlestick and sharp object injuries among Ethiopian HCWs. A total of 345 studies were identified from published and unpublished sources, with final selections of 22 eligible articles after removing duplications, inappropriate titles, and articles with no full text, quality problems, and no adjusted odds ratio. All eligible articles were considered in the forest plot to determine the pooled prevalence of needlestick and sharp object injuries. Regarding the distributions of the included studies, the majority were conducted in the Amhara region (3, 18, 19, 22–24, 38), followed by Oromia (27–30), SNNPRs (15, 16, 31), Addis Ababa (25, 26), and one each in Dire Dawa (33), Gambella (34), Sidama (35), Somali (36), and Tigray (32). All included studies used cross-sectional study designs.

Based on the findings of this study, the burden of needlestick and sharp objects injuries among healthcare workers in Ethiopia was high, and significantly influenced by needle stick recapping, the absence of workplace health and safety training, and the lack of routine precautionary measures.

The pooled prevalence of needlestick and sharp object injuries among healthcare workers in Ethiopia was 40.01 (95% CI: 34.72–45.30). This study finding was almost in line with the study conducted in Mongolia (38.4%) (39). However, it was higher than the studies conducted in Ghana (29.7%) (27), Nigeria (27%) (40), Thailand (23.7%) (41), Saudi Arabia (29.8%) (42), and Turkey (30.1%) (43). In contrast, the pooled prevalence was low than in studies conducted in Iran (67.8%) (27) and China (64.9%) (44).

The lack of routine precautionary measures among healthcare workers was positively associated with the occurrence of needlestick and sharp object injuries, which is consistent with the previous studies (27, 39, 45). Unsafe injection practices, such as reusing and recapping needles after injection, were identified by various studies conducted in Ethiopia. Poor waste segregation and disposal practices, a shortage of safety boxes, and inadequate awareness still remain major gaps in practice. Strengthening educational and training systems is thus important to enhance health care workers awareness of the standard precautions and protocols; a knowledge of which seems to be still far from adequacy in Ethiopia.

This study identified that needlestick recapping was another important factor for the occurrence of needlestick and sharp object injuries, a finding that is in accordance with previous studies in other countries (46, 47). This finding confirms the need to create awareness among healthcare workers on how to follow standard precautions in general and, specifically, on how to avoid needlestick recapping. If healthcare workers are required to administer an excessive number of injections, the likelihood of unsafe injections and ensuing needlestick and sharp object injuries may increase.

Training was another crucial factor in preventing the occurrence of needlestick and sharp object injuries among the healthcare workers in our study, which is consistent with the studies conducted in other countries (48, 49). According to this study, healthcare workers who had no occupational health and safety training were more exposed to needlestick and sharp object injuries compared to their counterparts.

## Limitations

This study has limitations that need to be considered when interpreting the results. One of the major limitations was the cross-sectional nature of the included studies, which could not establish a temporal relationship between needlestick and sharp object injuries and the independent variables. This results in a high chance of selection and information biases at the sample selection stage. There is likelihood for confounders to influence the results, resulting in incorrect interpretations. The second limitation of the study was the presence of heterogeneity among studies. The third limitation was the existence of publication bias, as could be observed from the funnel plot and Egger's test result. Fourth, the included studies were those with an abstract those fully tractable from major databases, which could affect the inclusiveness of the study. The last limitation was the disproportionate distribution of the studies in regions of Ethiopia, with the majority originating from the northern parts of the country.

## Conclusions

According to this systematic review and meta-analysis, 40.5% of the healthcare workers in Ethiopia had experienced needlestick and sharp object injuries. The lack of occupational health and safety training, the lack of routine adherence to standard precautions, and the practice of needlestick recapping practices increased the odds of needlestick and sharp object injuries in Ethiopia. The impact of needlestick and sharp object injuries in Ethiopia extends beyond individual healthcare workers; it can also affect the productivity of the country. Therefore, it is essential to give adequate emphasis to these factors to minimize injuries, morbidity, and mortality due to needlestick and sharp object injuries. The implementation of workplace safety standards at health facilities, regular follow-ups, and on-the-job training programs are crucial to overcoming this challenge.

## Data Availability

The original contributions presented in the study are included in the article/Supplementary Material, further inquiries can be directed to the corresponding author.
